# Stress-Strain Relationship of Ca(OH)_2_-Activated Hwangtoh Concrete

**DOI:** 10.1155/2014/846805

**Published:** 2014-03-04

**Authors:** Keun-Hyeok Yang, Ju-Hyun Mun, Hey-Zoo Hwang

**Affiliations:** ^1^Department of Plant, Architectural Engineering, Kyonggi University, Suwon, Kyonggi-do 443-760, Republic of Korea; ^2^Department of Architectural Engineering, Graduate School, Kyonggi University, Suwon, Kyonggi-do 443-760, Republic of Korea; ^3^Department of Architecture, Mokpo National University, Jeonnam 534-729, Republic of Korea

## Abstract

This study examined the stress-strain behavior of 10 calcium hydroxide (Ca(OH)_2_)-activated Hwangtoh concrete mixes. The volumetric ratio of the coarse aggregate (*V*_agg_) and the water-to-binder (*W/B*) ratio were selected as the main test variables. Two *W/B* ratios (25% and 40%) were used and the value of *V*_agg_ varied between 0% and 40.0%, and 0% and 46.5% for *W/B* ratios of 25% and 40%, respectively. The test results demonstrated that the slope of the ascending branch of the stress-strain curve of Ca(OH)_2_-activated Hwangtoh concrete was smaller, and it displayed a steeper drop in stress in the descending branch, compared with those of ordinary Portland cement (OPC) concrete with the same compressive strength. This trend was more pronounced with the increase in the *W/B* ratio and decrease in *V*_agg_. Based on the experimental observations, a simple and rational stress-strain model was established mathematically. Furthermore, the modulus of elasticity and strain at peak stress of the Ca(OH)_2_-activated Hwangtoh concrete were formulated as a function of its compressive strength and *V*_agg_. The proposed stress-strain model predicted the actual behavior accurately, whereas the previous models formulated using OPC concrete data were limited in their applicability to Ca(OH)_2_-activated Hwangtoh concrete.

## 1. Introduction

With the increasing importance of leadership in energy and environmental design (LEED) certifications for buildings and reducing greenhouse gas (GHG) emissions, many concrete industry players are strongly focusing on minimizing the use of ordinary Portland cement (OPC). Although OPC has played a prominent role in building and infrastructure development as the basic component of concrete and mortar, the production of a ton of OPC is commonly associated with the following environmental issues [1–4]: (1) CO_2_ emissions of 0.8–0.9 ton, which is approximately 7% of the total GHG emissions into the earth's atmosphere; (2) very high energy consumption including 90–100 kWh of electric power and 3-4 GJ of thermal energy owing to the plasticity temperature exceeding 1300°C; (3) natural resource depletion including 180–190 kg of bituminous coal and 20–30 kg of crude oil. For these reasons, the development of concrete with nil or minimal cement has attracted significant attention since the late 1980s.

Hwangtoh has gained more interest recently as a source material for low-cement concrete, especially in Korea and China, because it is known to be eco-friendly, with high absorption capacity and self-purifying characteristics, as well as offering health benefits by virtue of emitting far infrared radiation [5–7]. Hwangtoh is primarily clay formed by the weathering of rocks and composed of more than 70% inorganic substances, some organic material, water, and air. Yang et al. [6, 7] established that the workability and compressive strength development in calcium hydroxie (Ca(OH)_2_)-activated Hwangtoh mortars are comparable to those of OPC mortars with the same mixing proportions. However, more extensive use of Hwangtoh concrete in structural engineering applications warrants precise evaluations of its inherent characteristics including mechanical properties, inelastic deformation behavior, and durability. In particular, the compressive stress-strain relations of concrete incorporating a new binder should definitely be evaluated for use in the rational analysis and design of structural elements because the cohesion between pastes and aggregate particles significantly influences the extent of increase in strain due to the applied load [8]. The stress-strain relations are typically understood through various experiments and formulated using simple equations. However, no such experimental observations on the stress-strain relations of Ca(OH)_2_-activated Hwangtoh concrete are available in existing literature.

The objective of the present study is to propose a simple and rational model for obtaining the nonlinear stress-strain curves for Ca(OH)_2_-activated Hwangtoh concrete under compression. To formulate the key factor determining the slopes of the ascending and descending branches of the curves, 10 concrete mixes were prepared with a range of water-to-binder (*W*/*B*) ratios by weight and fine aggregate-to-total aggregate ratios (*S*/*a*) by volume. The developed model was then compared with existing models [9–11] that were empirically derived from the test data on OPC concrete. Furthermore, the modulus of elasticity and strain at peak stress of Ca(OH)_2_-activated Hwangtoh concrete were compared with those of OPC concrete collected from a variety of specimens [12] with compressive strength in similar ranges.

## 2. Experimental Program

### 2.1. Materials

Hwangtoh acquired high pozzolanic reactivity as a result of its calcination at a temperature of 850°C using the Hoffman method. The calcinated Hwangtoh, which was used as a source material, was activated by 7.5% Ca(OH)_2_. This Hwangtoh contained less calcium oxide (CaO) but was rich in both silicon oxide (SiO_2_) and aluminium oxide (Al_2_O_3_) as inferred from Table 1. This indicates that the chemical composition of Hwangtoh is very similar to that of fly ash and/or metakaolin [13]. All dry powdered alkali activators were preblended with the source materials in dry form.

The measurement of the physical properties of materials revealed the specific gravity and specific surface area of Hwangtoh to be 2.8 and 3200 cm^2^/g, respectively. The specific gravity, fineness modulus, and water absorption of the sand (used as the fine aggregate) were 2.42%, 2.51%, and 1.7%, respectively, and those of the crushed granite (used as the coarse aggregate with a maximum size of 19 mm) were 2.6%, 6.75%, and 1.1%, respectively. The specific gravity and maximum particle sizes of Ca(OH)_2_ were 2.24 and 21.2 *μ*m, respectively.

### 2.2. Specimen Mix Characteristics

In general, with increase in the compressive strength of concrete, the initial slope of its stress-strain curve increases, whereas the descending branch of the curves after peak stress has a more rapidly decreasing slope [14]. Moreover, concrete displays higher modulus of elasticity (*E*_*c*_) and smaller strain (*ε*_0_) values at peak stress than do mortars and pastes, indicating that the volumetric ratio of the coarse aggregates (*V*_agg_) directly affects the stress-strain curve of concrete [15, 16]. Considering these critical factors, the 10 cementless concrete mixes using Ca(OH)_2_-activated Hwangtoh binder were prepared with a range of *W*/*B* and *S*/*a* ratios, as listed in Table 2. The selected *W*/*B* ratios were 25% and 40%, and within each of these ratios, the *S*/*a* ratio of the mixes for each *W*/*B* ratio varied from 30% to 100% in intervals of 15%. The concrete specimen with an *S*/*a* ratio of 100% indicates a mortar without coarse aggregates. As a result, *V*_agg_ ranged between 0% and 40.0%, and 0% and 46.5% for *W*/*B* ratios of 25% and 40%, respectively. The unit water content was fixed at 180 kg/m^3^ for all concrete mixes. To achieve workability of the mixes for casting, a polycarboxylate-based, high-range water-reducing agent was added with binder ratio by weight of 1.5% and 1.2% for concrete mixes with *W*/*B* ratios of 25% and 40%, respectively.

### 2.3. Testing

**Figure 1:**
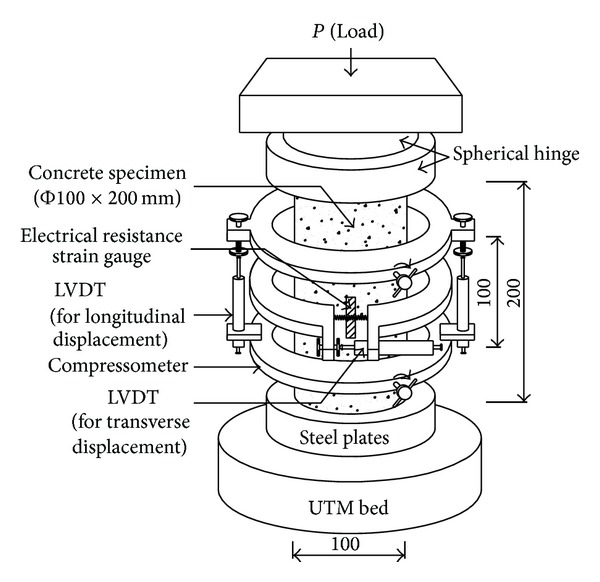
Test setup and instrumentation for test region (all dimensions are in mm).

All concrete specimens were mixed using a pan mixer of 0.35 m^3^ capacity and equipped with rubber wiper blades. The initial slump of fresh concrete was measured in accordance with the ASTM C143 provision [17]. Immediately after casting, all specimens used for plotting the stress-strain curve were cured at a constant temperature and relative humidity of 23 ± 2°C and 70 ± 5%, respectively, until testing at an age of 28 days. All steel moulds were removed after 1 day. The stress-strain curve was recorded using 100 × 200 mm cylindrical concrete specimens equipped with a compressor meter comprising linear variable differential transducers (LVDTs) of 50 mm capacity on both sides, as shown in Figure 1. The compressometer was combined with a convenient unbounded device for measuring transverse strain. Electrical resistance strain gages with gage length of 75 mm were also attached to the specimens. Prior to testing, both ends of each cylinder were leveled using a grinding machine. Concentric load was applied using a universal testing machine of 500 kN capacity, equipped with a closed-loop servocontrol system. Each specimen was preloaded to 20% of its compressive strength after which its position was adjusted based on strain gage and LVDT readings to achieve a concentric axial load. A spherical hinge was also positioned between the testing machine head and the specimens to minimize eccentricity in case of a large deformation. To obtain a complete stress-strain curve, a low strain rate of 2.0 × 10^−4^ per min was employed. Testing was continued until the final crushing of the concrete. All test data were captured by a data logger and stored automatically. Using the measured stress-strain curve, the *E*_*c*_ was calculated at 40% peak stress in accordance with the ASTM C469 provision [17].

## 3. Test Results and Discussion

### 3.1. General Behavior and Crack Propagation

**Figure 2:**
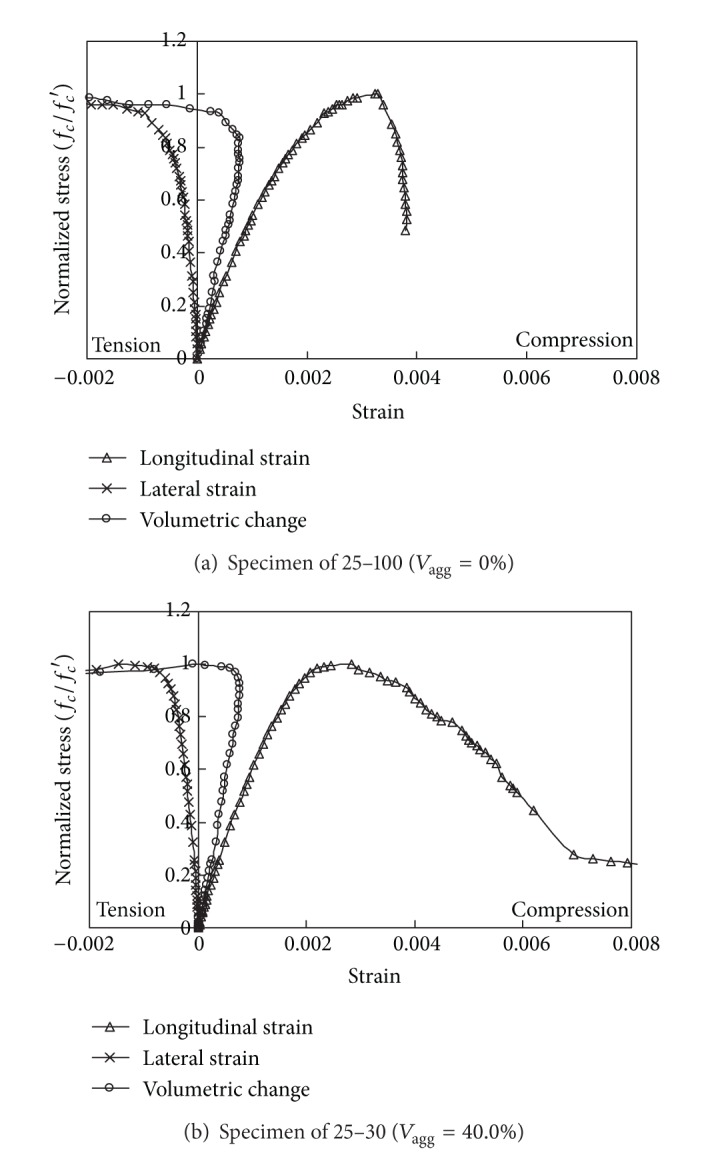
Typical strain behavior of Ca(OH)_2_-activated Hwangtoh concrete under axial compressive load.

Longitudinal cracking started at 50%–60% of the peak stress within the mid-height section of the specimen. The stress at which cracks developed was independent of *V*_agg_. As the applied load increased, the initial crack propagated toward the top and bottom surfaces of the specimens. As a result, the failure of specimens after peak stress was governed by the time when the longitudinal crack joined the top and bottom surfaces of the specimen. The width of the longitudinal cracks increased sharply at 80%–85% of the peak stress, which was accompanied by a rapid increase in the longitudinal and transverse strains, as shown in Figure 2. The rates of increase in those strains increased as *V*_agg_ decreased. With rapid increase in transverse strain, the volumetric change of the specimens started to shift from compression to tension. This threshold point was found to occur at approximately 80% and 95% of the peak stress for concrete mixes with *V*_agg_ of 0% and 40%, respectively. This implies that the rate of increase in transverse strain due to the opening of longitudinal cracks decreases as *V*_agg_ increases. After the peak stress, failure tended to occur along the longitudinal cracks, which commonly formed in the pastes between the aggregate particles. The drop in stress subsequent to peak stress was more rapid in concrete mixes with *V*_agg_ = 0% than those with *V*_agg_ = 40%. Figure 2 also shows that aggregate interlock action is expected along the longitudinal cracks.

### 3.2. Compressive Strength

**Figure 3:**
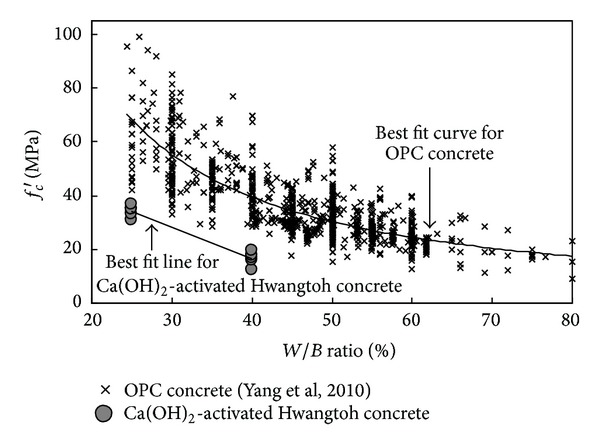
Effect of the water-to-binder ratio (*W*/*B*) on compressive strength (*f*_*c*_′).

Table 2 summarizes the test results including the compressive strength (*f*_*c*_′), *E*_*c*_, and strains of the Ca(OH)_2_-activated Hwangtoh concrete specimens. The value of *f*_*c*_′ increased by 1.89–2.52 times as the *W*/*B* ratio decreased from 40% to 25%. The rate of this increase also increased gradually as *V*_agg_ decreased. At both *W*/*B* ratios, the concrete specimens developed lower *f*_*c*_′ than did OPC concrete with no supplementary cementitious materials, as shown in Figure 3. However, the overall pattern of the decrease in *f*_*c*_′ corresponding to an increase in the *W*/*B* ratio was similar in both concrete types. The best-fit curve for *f*_*c*_′ confirmed that a *W*/*B* ratio not exceeding approximately 30% is required for structural applications of Ca(OH)_2_-activated Hwangtoh concrete. The *f*_*c*_′ of the test concrete also decreased as *V*_agg_ decreased (Table 2), at rates of 17% and 38% for *W*/*B* ratios of 25% and 40%, respectively, as the *S*/*a* ratio increased from 30% to 100%. The effect of *V*_agg_ on *f*_*c*_′ was more prominent for a higher *W*/*B* ratio. Higher coarse aggregate content leads to lower shrinkage and lower bleeding, resulting in less damage to the bond between the aggregate particles and pastes [8]. Hence, the coarse aggregate content is regarded as the secondary factor influencing the strength development in concrete, especially for one with a higher *W*/*B* ratio.

### 3.3. Stress-Strain Behavior

**Figure 4:**
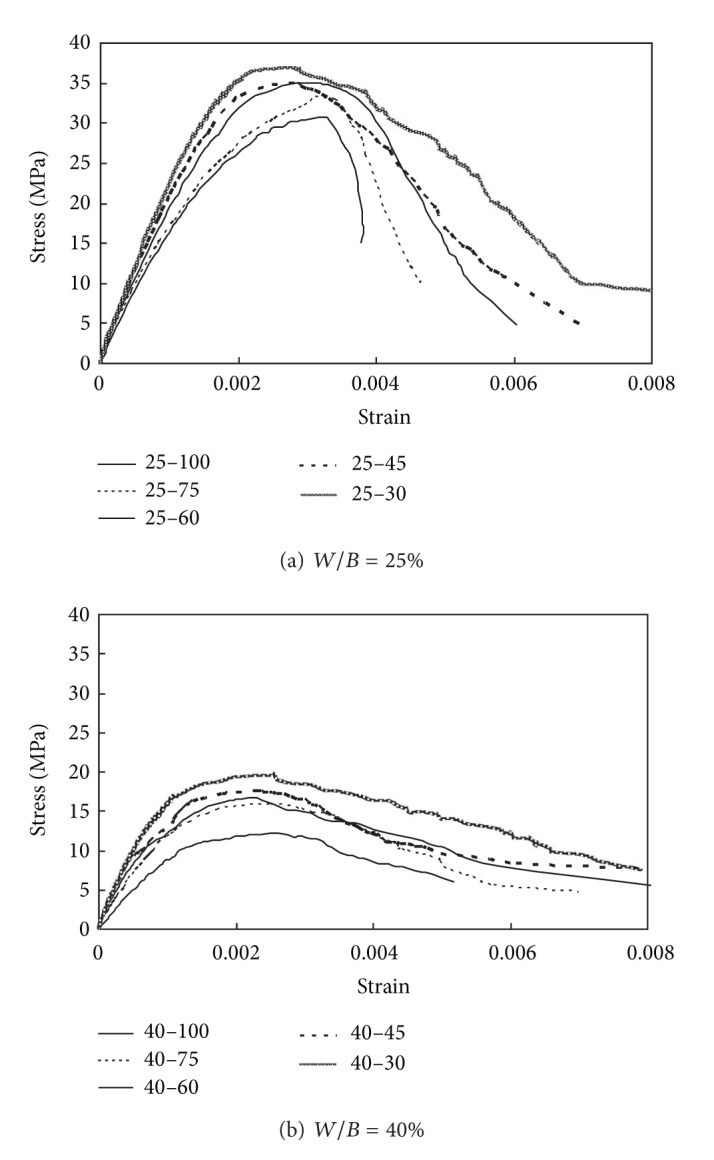
Stress-strain curves measured for Ca(OH)_2_-activated Hwangtoh concrete.

The stress-strain curves obtained for the concrete mixes are plotted in Figure 4. The shape of all curves was a second-degree parabola with its vertex at the peak stress point. However, the slopes of the ascending and descending branches of the curves heavily depended on the *f*_*c*_′ and *V*_agg_ values. Similar to observations on OPC concrete [9–11], the curves were almost linear up to approximately one-half of the peak stress point, showing that their initial slope increased as *f*_*c*_′ and/or *V*_agg_ increased. Normal-weight aggregates possess elastic modulus values that are 1.5–5.0 times higher than those of pastes [14], causing the initial slope of the curves to rise with increase in *V*_agg_. Additionally, the slope of the descending branch was steeper and less steep with increasing *f*_*c*_′ and *V*_agg_, respectively, though a slightly higher value of *f*_*c*_′ was obtained for the concrete mix with a higher *V*_agg_ value but the same *W*/*B* ratio. Overall, the descending branch of the curve for concrete mixes with *V*_agg_ = 0% (i.e., specimens 25–100 and 40–100) was very short compared to those for mixes with coarse aggregates. These trends were more pronounced for a *W*/*B* ratio of 25% than for 40%, leading to the inference that the mechanical interlocking of the coarse aggregate along the cracks relives the brittle fracture (as indicated by the descending branch) and improves the compressive strength of the concrete.

### 3.4. Modulus of Elasticity (*E*_*c*_)

**Figure 5:**
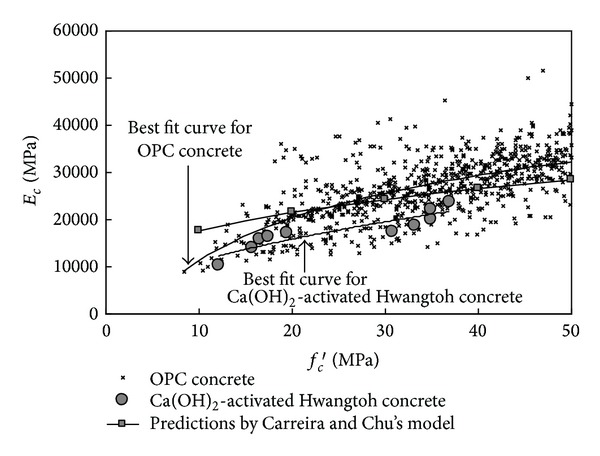
Modulus of elasticity (*E*_*c*_) as a function of compressive strength (*f*_*c*_′).

The relationship of *f*_*c*_′ and *E*_*c*_ of the Ca(OH)_2_-activated Hwangtoh concrete is shown in Figure 5. The figure also shows the test data for normal-weight OPC concrete with *f*_*c*_′ not exceeding 50 MPa, as well as predictions made by Carreira and Chu's model [9]. In their model, the unit weight (*w*_*c*_) of normal-weight concrete was assumed to be 2300 kg/m^3^. The *E*_*c*_ of the concrete was commonly lower than the best-fit curves determined for OPC concrete and predictions of Carreira and Chu's model [9]. The development of bond microcracks at the interfaces between pastes and aggregate particles accelerates the nonlinearity of the stress-strain curves [14], possibly causing the cohesion between them to be lower in Ca(OH)_2_-activated Hwangtoh binder than OPC.

**Figure 6:**
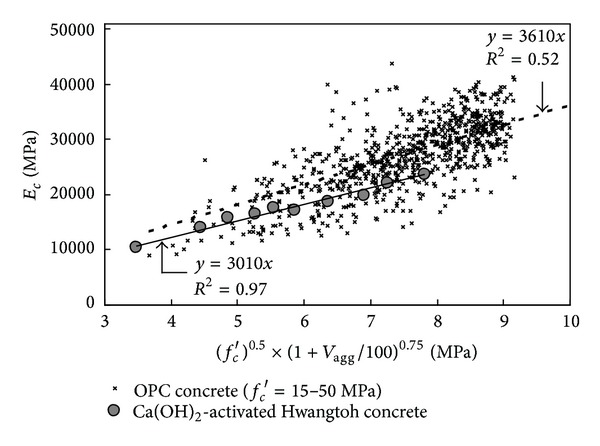
Regression analysis for modulus of elasticity (*E*_*c*_).

In general, the *E*_*c*_ of concrete can be expressed as a function of *f*_*c*_′ and *w*_*c*_ [14]. However, it was difficult to determine the effect of *w*_*c*_ on the *E*_*c*_ of the tested Ca(OH)_2_-activated Hwangtoh concrete because the variation in the *w*_*c*_ values (in the test) was very minimal at the same *W*/*B* ratios, ranging between 2419 and 2431 kg/m^3^, 2321 and 2335 kg/m^3^ for *W*/*B* ratios of 25% and 40%, respectively. Moreover, *E*_*c*_ was affected by *V*_agg_ (showing a slightly lower value in specimens with an *S*/*a* ratio of 60% than those with an *S*/*a* ratio of 45% at similar *f*_*c*_′) owing to the considerably higher modulus of elasticity in aggregates than in pastes when individually subjected to load. In addition, an increase in the continuous cracks joining the bond microcracks that developed at the interface between the pastes and aggregate particles leads to a faster rate of increase in strain [8]. To formulate a simple equation for *E*_*c*_, influential parameters such as *f*_*c*_′ and *V*_agg_ were repeatedly combined and adjusted through a trial-and-error approach until a relatively high correlation coefficient (*R* ^2^) was obtained. Based on a regression analysis of the test results, the *E*_*c*_ of Ca(OH)_2_-activated Hwangtoh concrete with *f*_*c*_′ < 40 MPa can be empirically expressed as follows (see Figure 6):

(1) $$\begin{array}{c} E_{c}=3010{\left(f_{c}^{\prime }\right)}^{0.5}{\left(1+\frac{V_{\text{agg}}}{100}\right)}^{0.75}\;\left(\text{MPa}\right). \end{array}$$

### 3.5. Strain at Peak Stress (*ε*_0_)

**Figure 7:**
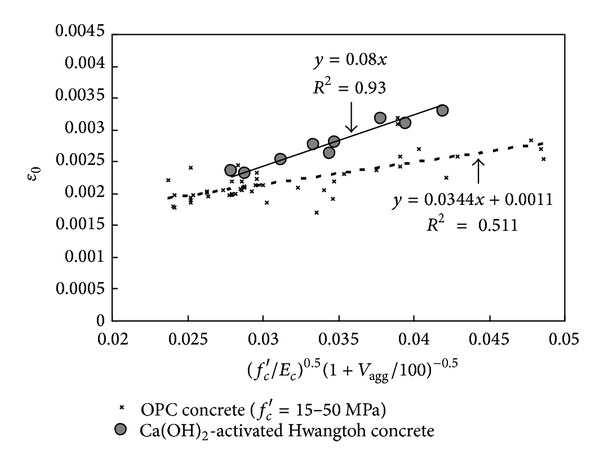
Regression analysis for strain (*ε*_0_) at peak stress.

MacGregor and Wight [14] established that *ε*_0_ of concrete increases with increase in *f*_*c*_′. This trend was also verified for the test case from the data listed in Table 2. As a result, the existent equations for *ε*_0_ have mostly been empirically developed only as a function of *f*_*c*_′. However, Ca(OH)_2_-activated Hwangtoh concrete demonstrated higher values of *ε*_0_ than did OPC concrete with the same *f*_*c*_′, as shown in Figure 7. These *ε*_0_ values also increased slightly as *V*_agg_ decreased because the increase in paste volume reduced the *E*_*c*_ and accelerated the nonlinearity of the stress-strain curves, especially after the development of longitudinal cracks. However, none of the predicted equations [9–11] consider the effect of *V*_agg_ on the strain although the former is evidently the secondary factor causing the increasing strain rate. Based on a regression analysis of the test results, the *ε*_0_ of Ca(OH)_2_-activated Hwangtoh concrete can be written as follows (see Figure 7):

(2) $$\begin{array}{c} \varepsilon_{0}=0.08{\left(\frac{f_{c}^{\prime }}{E_{c}}\right)}^{0.5}{\left(1+\frac{V_{\text{agg}}}{100}\right)}^{-0.5}. \end{array}$$

## 4. Mathematical Equations for Stress-Strain Relations

Figure 4 confirms that the compressive stress-strain curve of Ca(OH)_2_-activated Hwangtoh concrete is parabolic and hump-shaped with its vertex at the peak stress point. This physically means that the tangential modulus of elasticity has the maximum value at the origin, gradually decreases to zero at the peak stress point, and becomes negative through the descending branch of the curve. In this study, the same assumption and the following nonlinear equation [9, 11, 12] were applied for generating a complete curve:

(3) $$\begin{array}{c} Y=\frac{\left(\beta_{1}+1\right)X}{X^{\beta_{1}+1}+\beta_{1}}, \end{array}$$

where *Y*( = *f*_*c*_/*f*_*c*_′) is the normalized stress, *X*( = *ε*_*c*_/*ε*_0_) is the normalized strain, and *f*_*c*_ is the concrete stress corresponding to strain *ε*_*c*_. Equation (3) gives the following physical meanings: (1) *Y* = 0 when *X* = 0, representing the origin point; (2) *Y* = 1 when *X* = 1, representing the peak stress point; (3) *df*_*c*_/*dε*_*c*_ = 0 when*X* = 1 or at the peak stress point. Hence, the factor *β*_1_ in (3) is a key parameter for determining the slopes of the ascending and descending branches of the curve with its values commonly differing in each branch. To determine the slope of the ascending branch, *E*_*c*_ was selected as the reference parameter. Because *E*_*c*_ is defined as a secant modulus joining the origin and 40% of the peak stress, substituting it in (3) results in the following equation for determining *β*_1_ for the ascending branch:

(4) $$\begin{array}{c} 0.4{\left(X_{a}\right)}^{\beta_{1}+1}+\left(0.4-X_{a}\right)\beta_{1}-X_{a}=0\;\text{for}\;\varepsilon_{c}\leq \varepsilon_{0}, \end{array}$$

where *X*_*a*_ = 0.4*f*_*c*_′/*E*_*c*_ *ε*_0_.

In contrast, some reviews [12, 18] concluded that the secant modulus joining the origin and 50% of the peak stress can be regarded as an adequate reference point to derive the slope of the descending branch. Hence, the present study formulated an equation for determining *β*_1_ for the descending branch using the secant modulus at 0.5 *f*_*c*_′ as the reference point, as follows:

(5) $$\begin{array}{c} {\left(X_{d}\right)}^{\beta_{1}+1}+\left(1-2X_{d}\right)\beta_{1}-2X_{d}=0\;\text{for}\;\varepsilon_{c}>\varepsilon_{0}, \end{array}$$

where *X*_*d*_ = *ε*_0.5_/*ε*_0_ and *ε*_0.5_ is the strain corresponding to 0.5 *f*_*c*_′ after peak stress. The values of *β*_1_ in the nonlinear equations (4) and (5) can be calculated through numerical analysis for the given material properties of *E*_*c*_, *ε*_0_, and *ε*_0.5_.

### 4.1. Strain at 50% of Peak Stress in Descending Branch (*ε*_0.5_)

**Figure 8:**
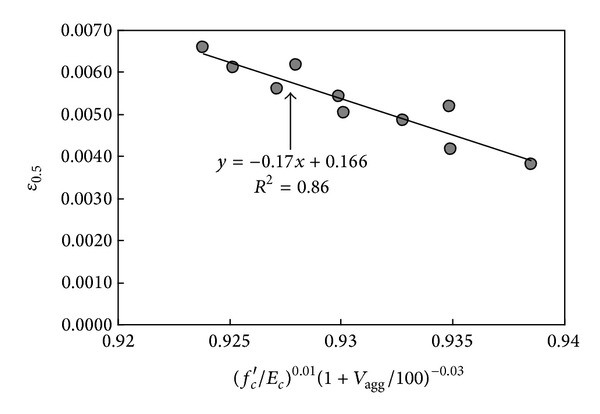
Regression analysis for *ε*_0.5_ of Ca(OH)_2_-activated Hwangtoh concrete.

The slope of the descending branch of the curve depends on *f*_*c*_′ and *V*_agg_, as inferred from Table 1. A regression analysis was conducted using these parameters (as shown in Figure 8) similar to the procedure employed for *ε*_0_ to propose a simple equation for *ε*_0.5_, yielding the following equation:

(6) $$\begin{array}{c} \varepsilon_{0.5}=-0.17\left[{\left(\frac{f_{c}^{\prime }}{E_{c}}\right)}^{0.01}{\left(1+\frac{V_{\text{agg}}}{100}\right)}^{-0.03}\right]+0.166. \end{array}$$

### 4.2. Key Parameter (*β*_1_)

**Figure 9:**
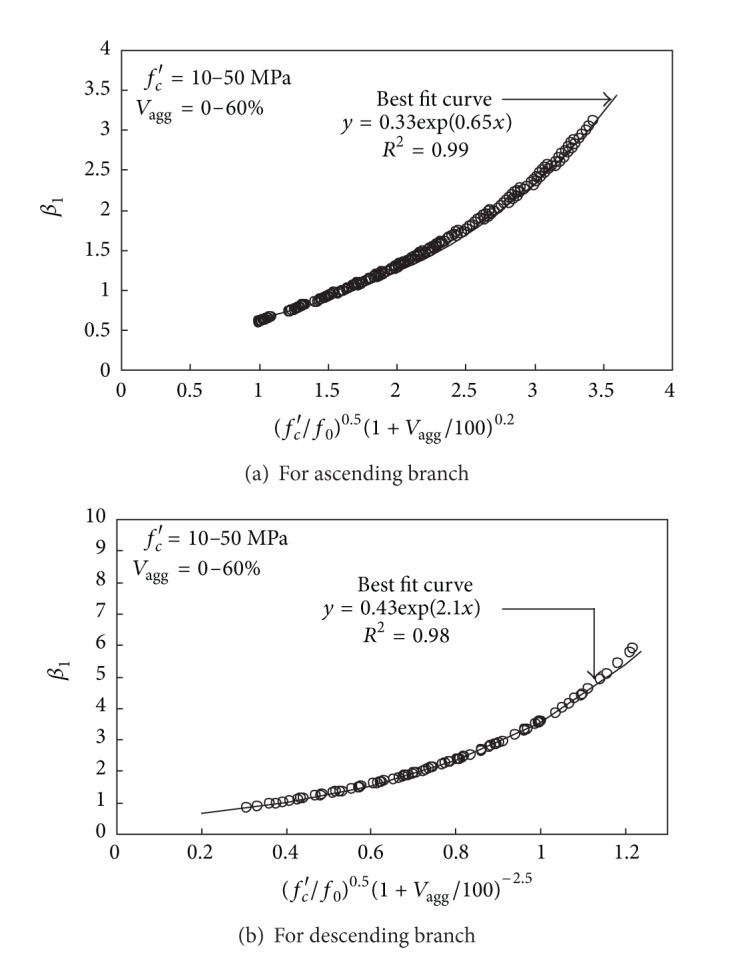
Formulation of *β*_1_ through numerical analysis.

An analytical parametric study was conducted to formulate *β*_1_, which determines the slopes of the ascending and descending branches. For given *f*_*c*_′ and *V*_agg_, the material properties of *E*_*c*_, *ε*_0_, and *ε*_0.5_ were calculated using (1), (2), and (6), respectively. Subsequently, the two non-linear equations ((4) and (5)) were solved using the Newton-Raphson method. Considering the practical strength development in Ca(OH)_2_-activated Hwangtoh concrete, the variables *f*_*c*_′ and *V*_agg_ in the analytical parametric study were selected to range between 10 and 50 MPa and 0% and 60%, respectively. Finally, the analytically obtained results were statistically optimized to drive the following *β*_1_ equations for the ascending branch (Figure 9(a)) and the descending branch (Figure 9(b)):

(7) $$\begin{array}{c} \beta_{1}=0.33\exp\left\lfloor 0.65{\left(\frac{f_{c}^{\prime }}{f_{0}}\right)}^{0.5}{\left(1+\frac{V_{\text{agg}}}{100}\right)}^{0.2}\right\rfloor \;\text{for}\;\varepsilon_{c}\leq \varepsilon_{0} \end{array}$$

(8) $$\begin{array}{c} \beta_{1}=0.43\exp\left\lfloor 2.1{\left(\frac{f_{c}^{\prime }}{f_{0}}\right)}^{0.5}{\left(1+\frac{V_{\text{agg}}}{100}\right)}^{-2.5}\right\rfloor \;\text{for}\;\;\varepsilon_{c}>\varepsilon_{0}, \end{array}$$

where *f*_0_ (= 10 MPa) is the reference value for the compressive strength of concrete. A higher value of *β*_1_ in (3) produces larger slopes of the ascending and descending branches. Hence, an increase in the value of *V*_agg_ in (7) and (8) results in a higher value of *E*_*c*_ and less steep slope of the descending branch, as observed in Figure 4.

In summary, the compressive stress-strain relations for unconfined Ca(OH)_2_-activated Hwangtoh concrete can be generalized as follows:

(9) $$\begin{array}{c} f_{c}=\left[\frac{\left(\beta_{1}+1\right)\left(\varepsilon_{c}/\varepsilon_{0}\right)}{{\left(\varepsilon_{c}/\varepsilon_{0}\right)}^{\beta_{1}+1}+\beta_{1}}\right]f_{c}^{\prime }, \end{array}$$

where *ε*_0_ and *β*_1_ are obtained using (2) and (7) or (8), respectively. The stress-strain relations proposed for the concrete need to be reexamined for strengths of more than 40 MPa because these equations have been derived using the limited test data pertaining to the present study.

## 5. Comparisons of Model and Test Results

**Figure 10:**
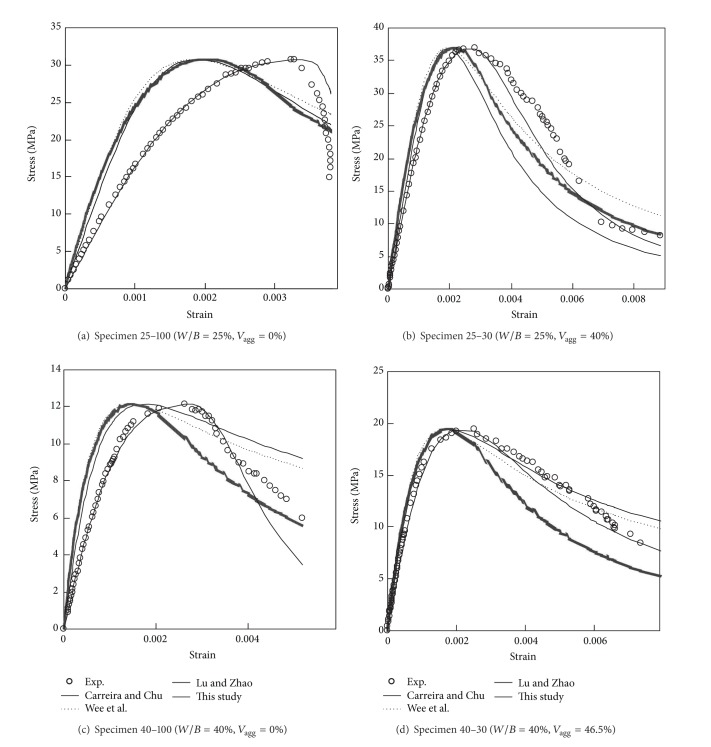
Typical comparisons of predicted and measured stress-strain curves for various *W*/*B* ratio and *V*_agg_ values.

Figure 10 shows the typical comparisons of the predicted and measured curves for different *W*/*B* ratios and *V*_agg_ values. The figure also displays the curves pertaining to previous models [9–11] that were empirically formulated using OPC test data.  Table 3 lists the mean (*γ*_*e*,*m*_) and standard deviation (*γ*_*e*,*s*_) of the normalized root-mean-square errors (NRMSE) calculated for each stress-strain curve. In general, the previous models commonly overestimate the slope of the ascending branch and underestimate the value of *ε*_0_. This trend is more pronounced with the increase in *f*_*c*_′ and decrease in *V*_agg_. Furthermore, the equations proposed by [9] and Wee et al. [11] overestimate the stress in the descending branch, particularly for concrete with low *V*_agg_. Consequently, the previous models yield high values for *γ*_*e*,*m*_ (>0.238) and *γ*_*e*,*s*_ (>0.050) and are limited in their applicability to Ca(OH)_2_-activated Hwangtoh concrete, and the error associated with them gradually increases as *V*_agg_ decreases.

In contrast to the previous models for OPC concrete, the predictions obtained from the proposed model for Ca(OH)_2_-activated Hwangtoh concrete are in better agreement with test results, showing relatively higher accuracy for both ascending and descending branches, judging from the considerably lower NRMSE values (0.091 and 0.039 for *γ*_*e*,*m*_ and *γ*_*e*,*s*_, resp.). These results are consistent with the actual responses: in particular, they accurately reflect the effect of *V*_agg_ on *ε*_0_ and the slopes of the ascending and descending branches. Hence, the proposed model is useful for accurately evaluating the compressive stress-strain behavior of low- and medium-strength Ca(OH)_2_-activated Hwangtoh concrete.

## 6. Conclusions

The present study examined the stress-strain behavior of Ca(OH)_2_-activated Hwangtoh concrete with different *W*/*B* and *V*_agg_ values. The *E*_*c*_ and *ε*_0_ values of the specimens were compared to those of OPC concrete compiled by Yang et al. Based on the experimental data, a simple equation was established to reasonably predict the stress-strain relations of Ca(OH)_2_-activated Hwangtoh concrete under compression, though further verification is required for strengths of more than 40 MPa because of the limited data used for empirical fitting. From the experimental and comparative observations, the following conclusions may be drawn.

1. For the same *W*/*B*, the *f*_*c*_′ of Ca(OH)_2_-activated Hwangtoh concrete was commonly lower than that of OPC concrete; however, the test concrete gained strength development comparable to that of structural concrete at a *W*/*B* less than 30%.
2. With decrease in *V*_agg_, the slope of the ascending branch of the stress-strain curve and *f*_*c*_′ decreased, whereas *ε*_0_ and the declining slope of the descending branch of the curve increased. This observation was more pronounced as the *W*/*B* ratio increased.
3. Compared to OPC concrete with the same *f*_*c*_′, Ca(OH)_2_-activated Hwangtoh concrete had lower *E*_*c*_ and larger *ε*_0_, showing greater discrepancy as *V*_agg_ decreased. Hence, the *E*_*c*_ and *ε*_0_ of Ca(OH)_2_-activated Hwangtoh concrete could be formulated as a function of *f*_*c*_′ and *V*_agg_.
4. Previous models formulated using OPC concrete data revealed limitations in their applicability to Ca(OH)_2_-activated Hwangtoh concrete, whereas the proposed stress-strain model predicted the actual behavior quite accurately, as evidenced by the much lower NRMSE values.

## Figures and Tables

**Table 1:** Chemical composition of Hwangtoh (% by mass).

SiO_2_	Al_2_O_3_	Fe_2_O_3_	CaO	MgO	K_2_O	Na_2_O	TiO_2_	MnO	LOI*
52.50	32.90	4.31	0.40	4.37	1.50	2.00	0.69	0.24	1.09

*Loss on ignition.

**Table 2:** Details of Ca(OH)_2_-activated Hwangtoh concrete mix proportions and summary of test results.

Group	Specimen*	*W*/*B* (%)	*S*/*a* (%)	*V*_agg_ (%)	Unit weight (kg/m^3^)					*w*_*c*_ (kg/m^3^)	*f*_*c*_′ (MPa)	*E*_*c*_ (MPa)	*ε*_0_	*ε*_0.5_
					*W*	*B*	*S*	*G*	*S*_*p*_					
I	25–100	25	100	0.0	180	720	1469	0	10.8	2419	30.8	17468	0.0033	0.0038
	25–75		75	14.3			1102	372		2424	33.1	18563	0.0031	0.0042
	25–60		60	22.9			882	595		2426	35.0	19873	0.0032	0.0048
	25–45		45	31.4			661	818		2429	35.0	22040	0.0028	0.0054
	25–30		30	40.0			441	1041		2431	36.9	23607	0.0028	0.0062

II	40–100	40	100	0.0	180	450	1709	0	5.4	2321	12.2	10241	0.0026	0.0052
	40–75		75	16.6			1281	432		2326	15.7	13822	0.0025	0.0050
	40–60		60	26.6			1025	691		2329	16.6	15737	0.0023	0.0056
	40–45		45	36.6			769	951		2332	17.5	16382	0.0023	0.0061
	40–30		30	46.5			513	1210		2335	19.5	17043	0.0024	0.0066

*W*/*B*: water-to-binder ratio by weight, *S*/*a*: fine aggregate-to-total aggregate ratio by volume, *V*_agg_: volumetric ratio of coarse aggregate, *W*: water, *B*: binder, *S*: fine aggregate, *G*: coarse aggregate, *S*_*p*_: polycarboxylate-based, high-range water-reducing admixture, *w*_*c*_: dry density of concrete, *f*_*c*_′: compressive strength, *E*_*c*_: modulus of elasticity, *ε*_0_: strain at peak stress, and *ε*_0.5_: strain corresponding to 0.5*f*_*c*_′ after the peak stress.

*In specimen names, the first and second parts refer to the *W*/*B* ratio and *S*/*a* ratio, respectively, for example, 25–75 refers to Ca(OH)_2_-activated Hwangtoh concrete mix with *W*/*B* and *S*/*a* ratios of 25% and 75%, respectively.

**Table 3:** Statistical values of NRMSEs calculated by comparing stress-strain curves of various models to experimental data.

Error coefficients*	Prediction models			
	Carreira and Chu [9]	Wee et al. [11]	Lu and Zhao [10]	This study
γ_*e*,*m*_	0.238	0.254	0.259	0.091
γ_*e*,*s*_	0.075	0.072	0.050	0.039

*γ_*e*,*m*_ and γ_*e*,*s*_ refer to the mean and standard deviation, respectively, of the NRMSE calculated for each specimen using the following equation:

NRMSE = (1/(*f*_*c*_)_*m*_)∑[((*f*_*c*_)_Exp_−(*f*_*c*_)_Pre._)^2^/*n*]^1/2^, where (*f*_*c*_)_*m*_ is the mean of the measured stress, (*f*_*c*_)_Exp_ and (*f*_*c*_)_Pre_ are experimental and predicted stresses, respectively, and *n* is the number of measured points.
